# Meeting Death

**Published:** 1873-02

**Authors:** 


### MEETING DEATH

With desperation, with defiance, with pride, or haughtiness or dignity, or Christian resignation, depends on the life-char acter of the man.

Paul, with a steady courage, with a wise appreciation o an event so momentous, declared, " I am now ready to b offered; I have kept the faith; I look for my crown from the hands of the great Judge of all." The infidel Hume, the great historian, the wonderful trifler and buffoon, could think of nothing better than to ask for a pack of cards to while away the time on the last day of his life. The great Napoleon, while dying, called for his field-marshal's cloak and boots. Cæsar stood proudly erect, and throwing his gown across his breast and over his left shoulder, waited for the fatal stroke of the stiletto. Maria Theresa, of Austria, just before she expired said, "I could go to sleep, but I will not allow death to surprise me in my sleep."

"See in what peace a Christian can die," was the exclamation of Addison.

They can meet death with the most calmness and composure, who have lived regular, calm, and peaceful lives, enabling the human machine to wear out equally in all its parts, ceasing its operations without a shock or jar, and as gently as dies the ember on the hearth, or as the summer evening passes into the shades of night.

